# Supplementary material for: Extending the Range of Sizes of Monodisperse Core‐Shell Hydrogel Capsules from Composite Jet Breakup by Combined Electrical and Mechanical Actuation
Source: Adv Sci (Weinh). 2025 Jul 24;12(38):e05501. doi: 10.1002/advs.202505501 (PMC12520576; doi:10.1002/advs.202505501)
Supplement: Supplementary file 1 — Supporting Information [file ADVS-12-e05501-s001.docx]

Supporting Information

Extending the range of sizes of monodisperse core-shell hydrogel capsules from composite jet breakup by combined electrical and mechanical engineering

Lucas Suire, Anirban Jana, Pierre Nassoy*, Amaury Badon*

**Movie S1**

Bright-field video capturing the natural fragmentation of the composite jet without excitation and electric potential. Scale bar, 1 mm.

**Movie S2**

Bright-field video of the fragmentation of the composite jet for different excitation frequencies and without electric potential. Scale bar, 1 mm.

**Movie S3**

(Left) Bright-field video of the fragmentation of the composite jet at an excitation frequency of 1100 Hz and applied voltages of 2000 V and 3000 V. (Right) Zoomed-in slow-motion view of the filament rupture. Scale bar, 1 mm.

**Figure S1**

**Figure S1**. **Measurement of the fragmentation length.** (A) Video rotation and binarization by using an intensity threshold to distinguish the jet from the background. (B) Pixel intensity projection along the z-axis. Each droplet corresponds to a peak. The intensity between the peaks of the droplets prior to breakup is non-zero due to the presence of the filament. After breakup, the absence of the filament results in zero intensity between the drops. The red arrow indicates the first zero of this intensity projection. (C) Position of the first zero of the intensity projection as a function of time. The red cross represents the local maximum of the first zero position, corresponding to the fragmentation length. The code uses the find_peaks function from the SciPy library to detect this local maximum. The fragmentation length is then determined by averaging this value over more than 100 fragmentation events and adding the distance between the injector and the top of the camera's field of view.


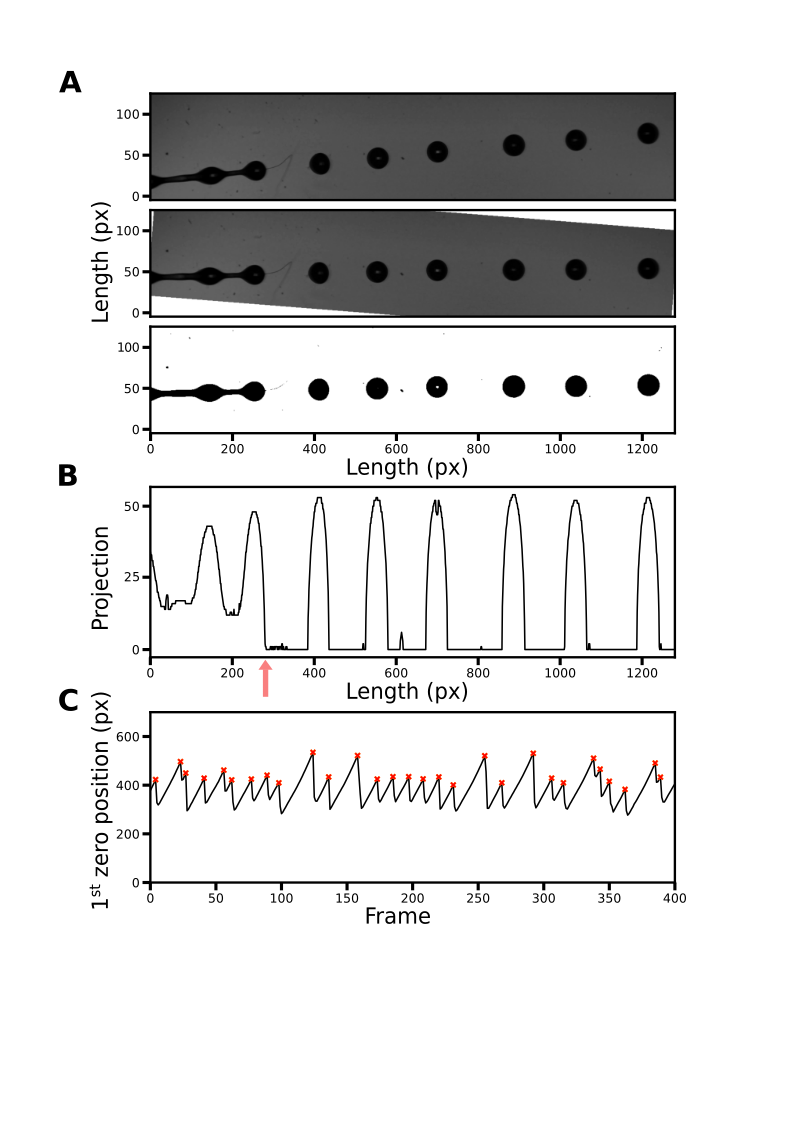


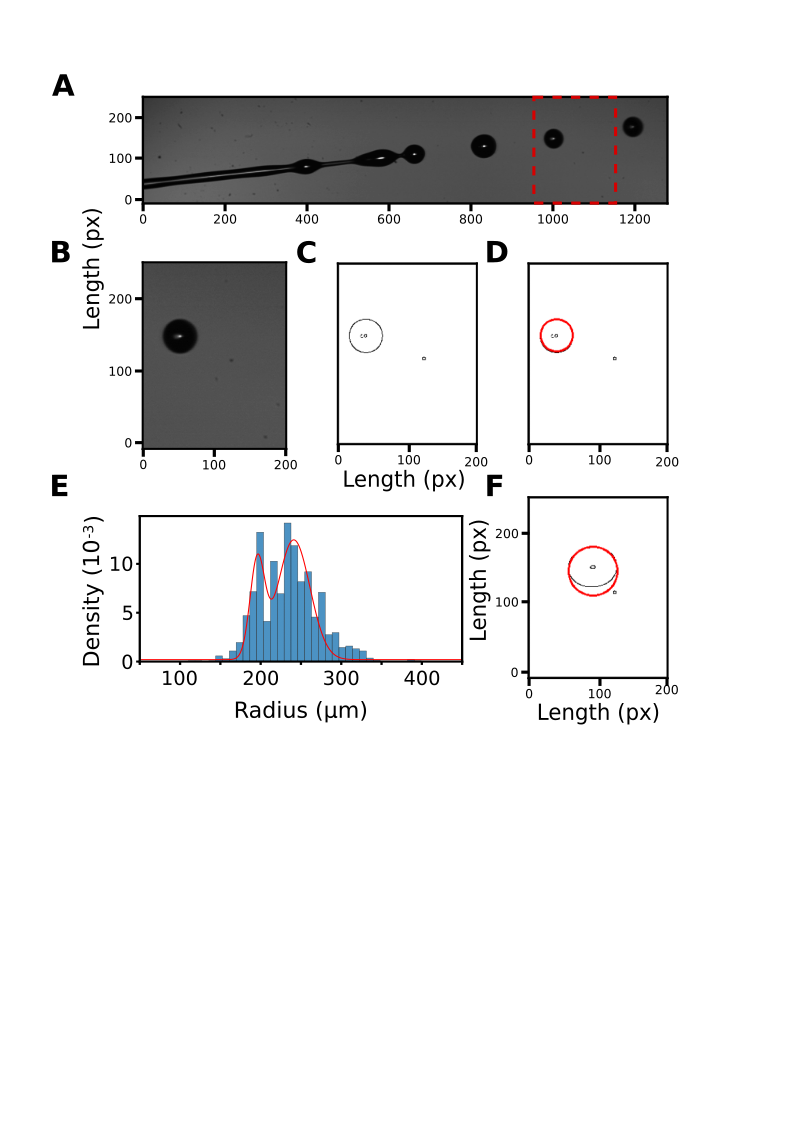
**Figure S2**

**Figure S2. Measurement of drops radius.** (A-B) Selection of an area of interest of 200 pixels (1700 µm^2^) in each video frame, starting 1000 µm downstream from the jet fragmentation spot. (C) Detecting drop contours using a Canny filter based on intensity gradients (Gaussian filter, sigma=1, and threshold values = 40 (low) and 60 (high)). This gradient thresholding ensures that out-of-focus droplets with blurred contours are excluded from detection. (D) Detecting drop radii using a circular Hough transform (minimum threshold=17 µm, maximum threshold= 850 µm). (E) Histogram of drop radius distribution with a Gaussian fit to derive the average radius and standard deviation. (F) Example of an oscillating drop with an elliptical shape. In this case, our algorithm detects both the minor and major radii, leading to an artificial increase in the standard deviation of the distributions.

**
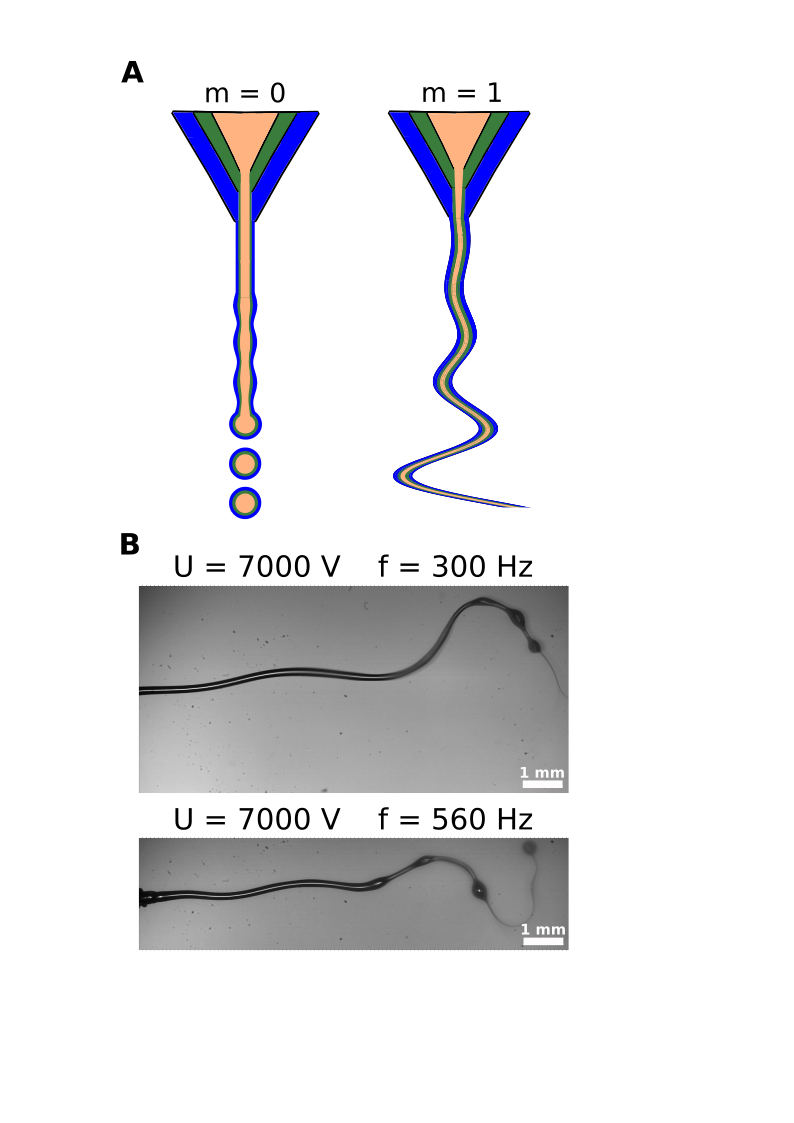
Figure S3**

**Figure S3. Whipping of the jet**. (A) Illustration of two instability modes: the Plateau-Rayleigh instability (m=0) and the whipping instability (m=1). (B) Whipping instability at U = 7000 V for two different excitation frequencies: f = 300 Hz and f = 560 Hz


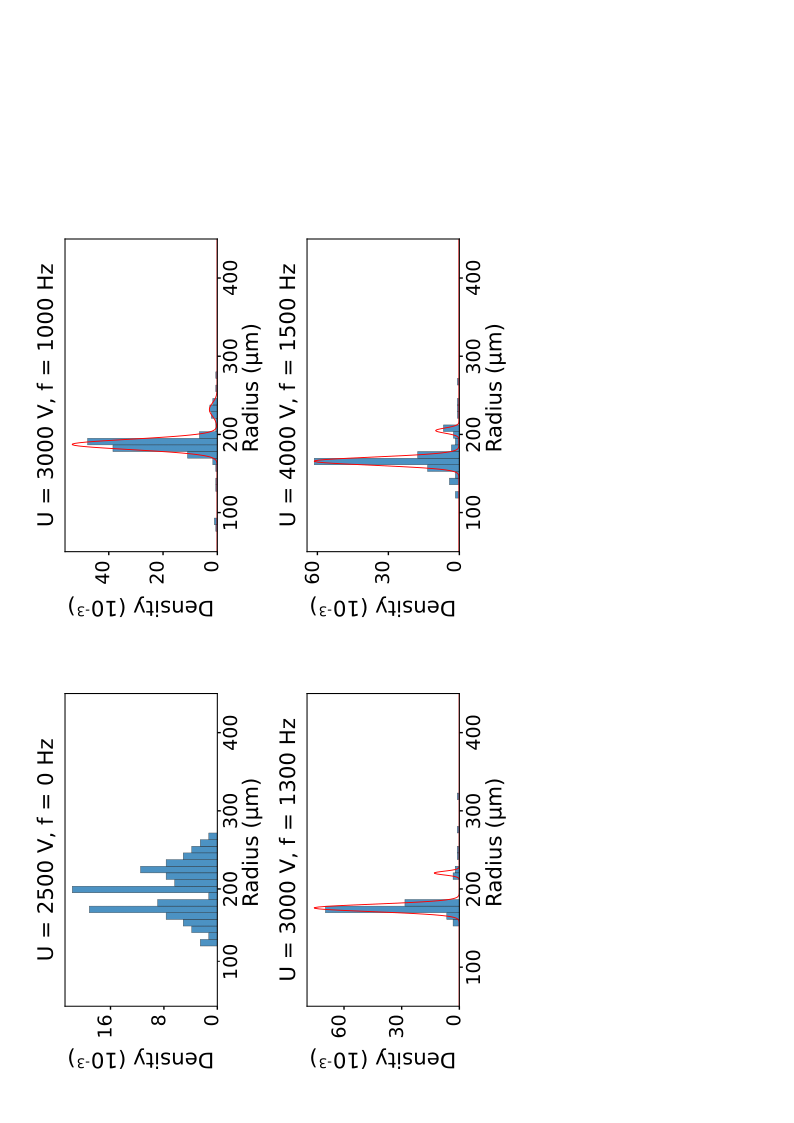


**Figure S4**

**Figure S4.** **Capsules sizes**. Histogram of capsules radius distribution with a Gaussian fit for different values of the electric potential U. Statistical values of the fits are provided in Table S1. Encapsulation experiments were performed with a concentration of 500 000 cells/mL.

**Table S1**

| **2500 V** | Total distribution | Mean radius (µm) | Standard deviation (µm) |  |
| --- | --- | --- | --- | --- |
| **0 Hz** |  | 197.9 | 30.9 |  |
| N = 92 |  |  |  |  |

| **3000 V** | Total distribution | Mean radius (µm) | Standard deviation (µm) |  |
| --- | --- | --- | --- | --- |
| **1000 Hz** |  | 187.8 | 19.9 |  |
| N = 214 | Gaussian fit | μ (µm) | σ (µm) | $A_{n}$ |
| $R^{2}=0.989$ | 1^st^ gaussian | 187.2 | 6.5 | 0.0535 |
|  | 2^nd^ gaussian | 231.8 | 8.4 | 0.0028 |

| **3000 V** | Total distribution | Mean radius (µm) | Standard deviation (µm) |  |
| --- | --- | --- | --- | --- |
| **1300 Hz** |  | 180.9 | 21.7 |  |
| N = 108 | Gaussian fit | μ (µm) | σ (µm) | $A_{n}$ |
| $R^{2}=0.997$ | 1^st^ gaussian | 175.8 | 4.7 | 0.0756 |
|  | 2^nd^ gaussian | 220.3 | 2.4 | 0.0131 |

| **4000 V** | Total distribution | Mean radius (µm) | Standard deviation (µm) |  |
| --- | --- | --- | --- | --- |
| **1500 Hz** |  | 170.2 | 19.1 |  |
| N = 140 | Gaussian fit | μ (µm) | σ (µm) | $A_{n}$ |
| $R^{2}=0.992$ | 1^st^ gaussian | 165.8 | 5.1 | 0.0613 |
|  | 2^nd^ gaussian | 204.9 | 3.3 | 0.0099 |

**Table S1**. Mean radius and standard deviation of the capsule radius distribution, along with parameters of the multiple Gaussian fit.

**Table S2**

**Table S2.** Theoretical drop radii, corresponding experimental capsule radii, and their ratio. Crosslinked capsules are 0.85 times smaller than the droplets from they originated.

| Conditions | Theoretical drops radius (µm) | Experimental capsules radius (µm) | Ratio |
| --- | --- | --- | --- |
| 3000 V 1000 Hz | 222 | 187 | 0.84 |
| 3000 V 1300 Hz | 203 | 176 | 0.86 |
| 4000 V 1500 Hz | 194 | 166 | 0.86 |

**Figure S5**


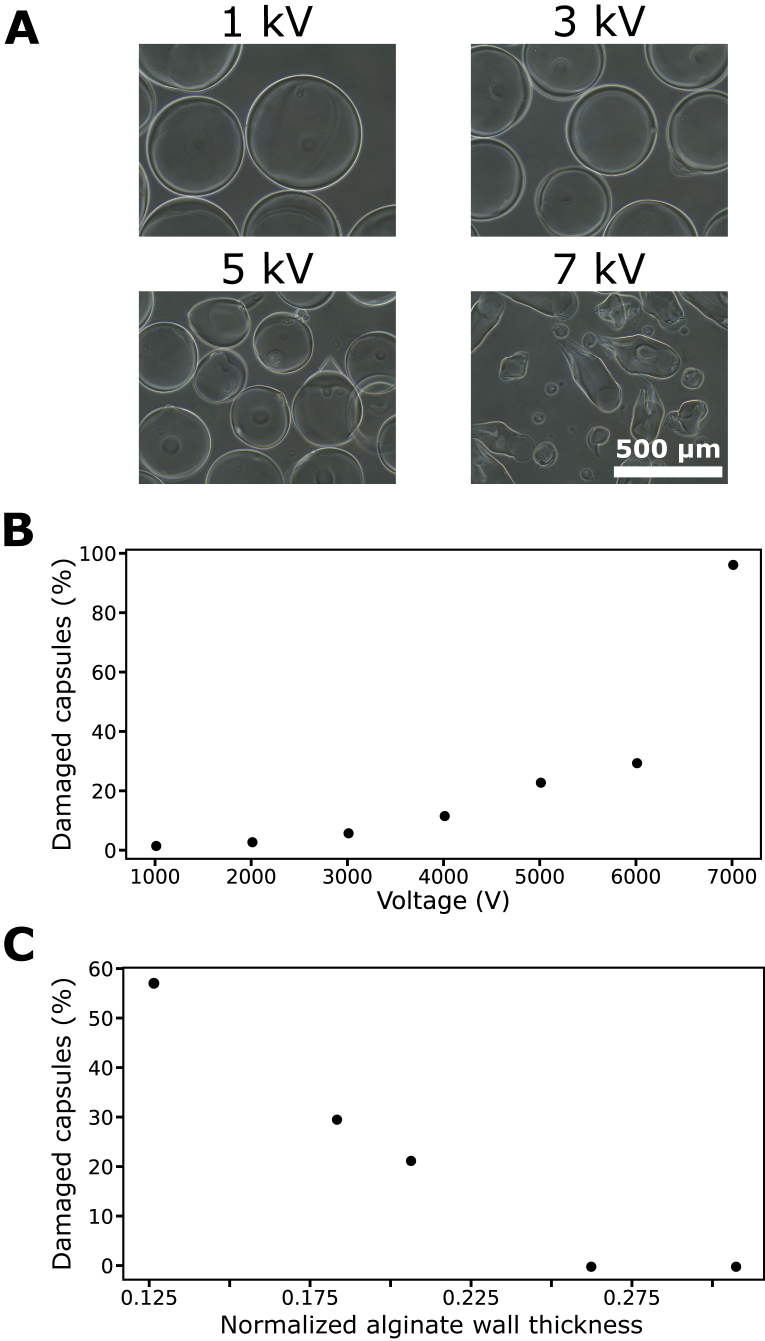


**Figure S5.** **Capsules disruption at high voltage.** (A) Micrographs of representative capsules in the calcium bath (*ie* after gelation) taken at *U*=1, 3, 5 and 7 kV and *f*=0 Hz. The average diameter of the capsules decreases as the voltage increases. At *U*=1 and 3 kV, the capsule are spherical and the thickness of the shell is homogeneous. At *U*=5 kV, shells become more heterogeneous. At *U*=7 kV, corresponding to the onset of visible whipping the integrity of the spherical capsule was impaired. (B) Plot of the percentage of damaged capsules as a function of voltage for a normalized alginate wall thickness of h = 0.183. The expression for the alginate wall thickness $e$, normalized by the capsule radius, is $h=\frac{e}{R_{cap}}=1-\left( \frac{r_{q}}{1+r_{q}} \right)^{\frac{1}{3}}$ with $r_{q}=\frac{V_{core}}{V_{shell}}=\frac{Q_{core}}{Q_{shell}}$ and Q the flow rate. In each condition, N>150. (C) Plot of the percentage of disrupted capsules as a function of normalized shell thickness for U = 6000 V.

**Table S3**

**Figure 1**

| **0 V** | Total distribution | Mean radius (µm) | Standard deviation (µm) |  |
| --- | --- | --- | --- | --- |
| **0 Hz** |  | 234.5 | 37.0 µm |  |
| N = 1625 | Gaussian fit | μ (µm) | σ (µm) | $A_{n}$ |
| $R^{2}=0.894$ | 1^st^ gaussian | 195.8 | 9.1 | 0.0098 |
|  | 2^nd^ gaussian | 241.1 | 20.0 | 0.0123 |

**Figure 2**

| **0 V** | Total distribution | Mean radius (µm) | Standard deviation (µm) |  |
| --- | --- | --- | --- | --- |
| **300 Hz** |  | 246.2 | 71.8 |  |
| N = 1331 | Gaussian fit | μ (µm) | σ (µm) | $A_{n}$ |
| $R^{2}=0.911$ | 1^st^ gaussian | 193.8 | 15.6 | 0.0094 |
|  | 2^nd^ gaussian | 240.6 | 10.9 | 0.0090 |
|  | 3^rd^ gaussian | 281.7 | 20.0 | 0.0063 |
| **0 V** | Total distribution | Mean radius (µm) | Standard deviation (µm) |  |
| **600 Hz** |  | 251.0 | 57.0 |  |
| N = 1654 | Gaussian fit | μ (µm) | σ (µm) | $A_{n}$ |
| $R^{2}=0.940$ | 1^st^ gaussian | 178.6 | 14.4 | 0.0042 |
|  | 2^nd^ gaussian | 256.4 | 18.6 | 0.0163 |
| **0 V** | Total distribution | Mean radius (µm) | Standard deviation (µm) |  |
| **1000 Hz** |  | 234.7 | 36.1 |  |
| N = 2040 | Gaussian fit | μ (µm) | σ (µm) | $A_{n}$ |
| $R^{2}=0.953$ | 1^st^ gaussian | 221.1 | 7.0 | 0.0428 |
| **0 V** | Total distribution | Mean radius (µm) | Standard deviation (µm) |  |
| **1400 Hz** |  | 230.8 | 38.9 |  |
| N = 2436 | Gaussian fit | μ (µm) | σ (µm) | $A_{n}$ |
| $R^{2}=0.981$ | 1^st^ gaussian | 199.4 | 4.8 | 0.0347 |
|  | 2^nd^ gaussian | 249.2 | 9.6 | 0.0164 |
| **0 V** | Total distribution | Mean radius (µm) | Standard deviation (µm) |  |
| **2000 Hz** |  | 270.8 | 48.0 |  |
| N = 1498 | Gaussian fit | μ (µm) | σ (µm) | $A_{n}$ |
| $R^{2}=0.878$ | 1^st^ gaussian | 182.1 | 5.5 | 0.0078 |
|  | 2^nd^ gaussian | 218.9 | 5.6 | 0.0025 |
|  | 3^rd^ gaussian | 250.4 | 6.8 | 0.0073 |
|  | 4^th^ gaussian | 284.2 | 15.0 | 0.0148 |
| **0 V** | Total distribution | Mean radius (µm) | Standard deviation (µm) |  |
| **2400 Hz** |  | 266.1 | 56.8 |  |
| N = 1391 | Gaussian fit | μ (µm) | σ (µm) | $A_{n}$ |
| $R^{2}=0.852$ | 1^st^ gaussian | 259.0 | 49.3 | 0.0080 |

**Figure 3**

| **0 V** | Total distribution | Mean radius (µm) | Standard deviation (µm) |  |
| --- | --- | --- | --- | --- |
| **1500 Hz** |  | 238.7 | 45.0 |  |
| N = 1940 | Gaussian fit | μ (µm) | σ (µm) | $A_{n}$ |
| $R^{2}=0.987$ | 1^st^ gaussian | 197.1 | 4.1 | 0.0279 |
|  | 2^nd^ gaussian | 245.2 | 7.5 | 0.0244 |
|  | 3^rd^ gaussian | 279.1 | 10.1 | 0.0056 |
| **3000 V** | Total distribution | Mean radius (µm) | Standard deviation (µm) |  |
| **1500 Hz** |  | 214.8 | 30.4 |  |
| N = 2326 | Gaussian fit | μ (µm) | σ (µm) | $A_{n}$ |
| $R^{2}=0.970$ | 1^st^ gaussian | 194.9 | 6.8 | 0.0350 |
|  | 2^nd^ gaussian | 244.1 | 8.0 | 0.0164 |
|  | 3^rd^ gaussian | 276.4 | 8.0 | 0.0020 |
| **5000 V** | Total distribution | Mean radius (µm) | Standard deviation (µm) |  |
| **1500 Hz** |  | 193.9 | 9.6 |  |
| N = 2103 | Gaussian fit | μ (µm) | σ (µm) | $A_{n}$ |
| $R^{2}=0.981$ | 1^st^ gaussian | 196.6 | 4.8 | 0.0756 |
| **6000 V** | Total distribution | Mean radius (µm) | Standard deviation (µm) |  |
| **1500 Hz** |  | 189.2 | 19.2 |  |
| N = 1795 | Gaussian fit | μ (µm) | σ (µm) | $A_{n}$ |
| $R^{2}=0.881$ | 1^st^ gaussian | 193.1 | 7.8 | 0.0503 |

**Figure 4**

| **0 V** | Total distribution | Mean radius (µm) | Standard deviation (µm) |  |
| --- | --- | --- | --- | --- |
| **800 Hz** |  | 242.4 | 22.3 |  |
| N = 1955 | Gaussian fit | μ (µm) | σ (µm) | $A_{n}$ |
| $R^{2}=0.996$ | 1^st^ gaussian | 240.9 | 6.8 | 0.0525 |
| **3000 V** | Total distribution | Mean radius (µm) | Standard deviation (µm) |  |
| **800 Hz** |  | 206.0 | 42.1 |  |
| N = 2286 | Gaussian fit | μ (µm) | σ (µm) | $A_{n}$ |
| $R^{2}=0.877$ | 1^st^ gaussian | 143.3 | 7.0 | 0.0161 |
|  | 2^nd^ gaussian | 230.9 | 15.2 | 0.0192 |
| **5000 V** | Total distribution | Mean radius (µm) | Standard deviation (µm) |  |
| **800 Hz** |  | 187.5 | 29.7 |  |
| N = 2066 | Gaussian fit | μ (µm) | σ (µm) | $A_{n}$ |
| $R^{2}=0.994$ | 1^st^ gaussian | 158.1 | 6.5 | 0.0282 |
|  | 2^nd^ gaussian | 215.6 | 4.7 | 0.0399 |
| **6000 V** | Total distribution | Mean radius (µm) | Standard deviation (µm) |  |
| **800 Hz** |  | 194.8 | 23.1 |  |
| N = 1041 | Gaussian fit | μ (µm) | σ (µm) | $A_{n}$ |
| $R^{2}=0.914$ | 1^st^ gaussian | 166.3 | 9.4 | 0.0075 |
|  | 2^nd^ gaussian | 203.3 | 9.3 | 0.0339 |
| **0 V** | Total distribution | Mean radius (µm) | Standard deviation (µm) |  |
| **2400 Hz** |  | 266.1 | 56.8 |  |
| N = 1391 | Gaussian fit | μ (µm) | σ (µm) | $A_{n}$ |
| $R^{2}=0.852$ | 1^st^ gaussian | 259.0 | 49.3 | 0.0080 |
| **3000 V** | Total distribution | Mean radius (µm) | Standard deviation (µm) |  |
| **2400 Hz** |  | 225.3 | 48.4 |  |
| N = 1700 | Gaussian fit | μ (µm) | σ (µm) | $A_{n}$ |
| $R^{2}=0.924$ | 1^st^ gaussian | 198.3 | 6.5 | 0.0190 |
|  | 2^nd^ gaussian | 241.7 | 18.0 | 0.0113 |
| **5000 V** | Total distribution | Mean radius (µm) | Standard deviation (µm) |  |
| **2400 Hz** |  | 200.9 | 39.4 |  |
| N = 1572 | Gaussian fit | μ (µm) | σ (µm) | $A_{n}$ |
| $R^{2}=0.862$ | 1^st^ gaussian | 205.2 | 14.2 | 0.0255 |
| **6000 V** | Total distribution | Mean radius (µm) | Standard deviation (µm) |  |
| **2400 Hz** |  | 180.9 | 43.2 |  |
| N = 1973 | Gaussian fit | μ (µm) | σ (µm) | $A_{n}$ |
| $R^{2}=0.959$ | 1^st^ gaussian | 166.6 | 6.6 | 0.0422 |

**Table S3**. Mean radius and standard deviation of the capsule radius distribution, along with parameters of the multiple Gaussian fits from the figures in the main article. Bin width = 8.5 µm.


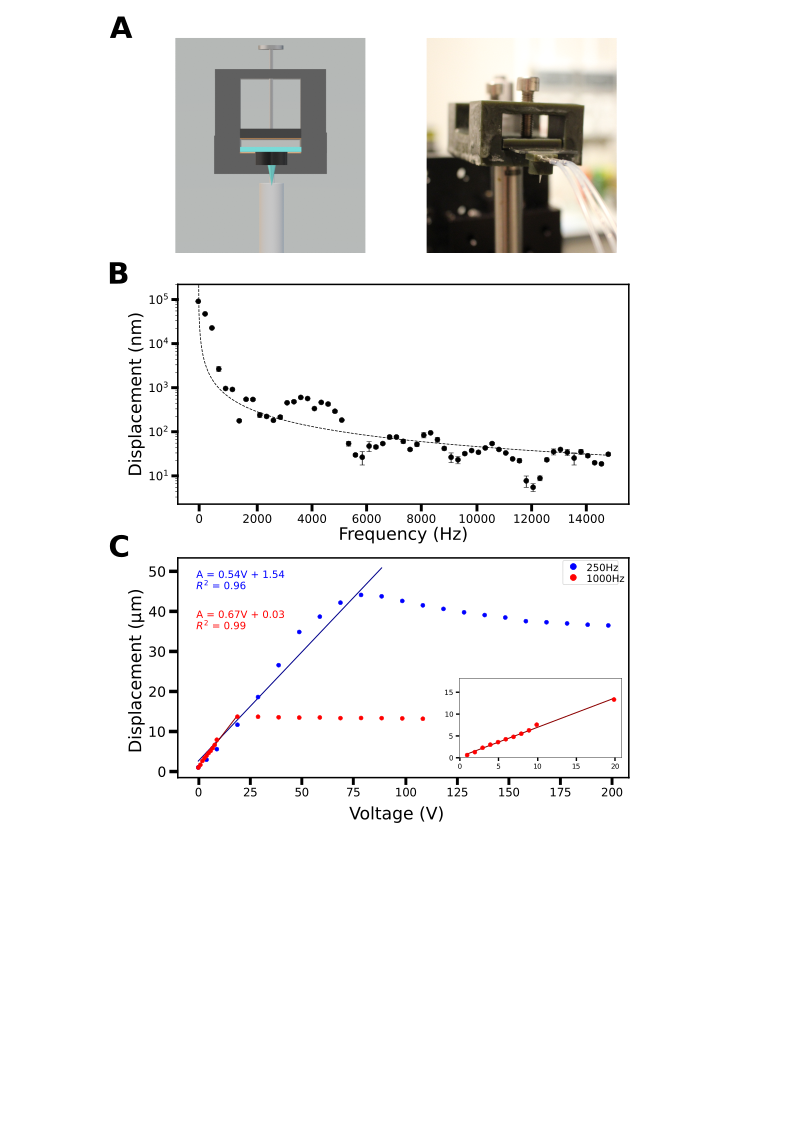
**Figure S6**

**Figure S6.** **Piezo-electric-induced excitation of the injector.** (A) 3D modelling and photography of the support and piezoelectric actuator. (B) Displacement of the piezoelectric actuator as a function of frequency *f* for a constant applied voltage of $U_{pp}=200 V$ measured with an interferometer (Attocube, IDS3010). (C) Displacement of the piezoelectric actuator as a function of the voltage $U_{pp}$ for two different frequencies: *f* = 250 Hz and *f* = 1000 Hz. In all the experiments, we chose a periodic voltage with a peak-to-peak amplitude of 6 V applied to the PZT resulting in a constant piezo oscillation of approximately 4 µm. Under these conditions, the bandwidth extends up to approximately 4000 Hz.

**Growth rate calculation and fragmentation length definition**

In the spatial linear description of the Plateau-Rayleigh instability, for an axial perturbation $A(t,z)=A_{0}e^{i(\omega t-kz)}$, with ω the angular frequency and k the wavenumber, ω is purely real, and k is complex. The growth rate is set by the imaginary part of the wavenumber, $Im\left\{ k \right\}$. By considering that, at the fragmentation length $z=L_{frag}$, the disturbance amplitude is equal to the radius of the jet, on obtains :

$A\left( z=L_{frag} \right)= A_{0}e^{Im\left\{ k \right\}L_{frag}}= R_{jet}$ (1)

Or, equivalently

$Im\left\{ k \right\}=\frac{1}{L_{frag}}\ln\left( \frac{R_{jet}}{A_{0}} \right)$ (2)

The growth rate corresponds to the inverse of the fragmentation length, normalized by a constant that depends logarithmically on the ratio of the jet radius to the initial excitation amplitude. Figure S4 illustrates the steps undertaken to determine the instability growth rate curve as a function of excitation frequencies with an alternative voltage of amplitude $U_{pp}=6 V$ applied to the piezoelectric actuator. Figure S7A displays the fragmentation length as a function of the excitation frequency at U_PP_=6V. $L_{frag}$ was obtained using the described in Fig. S1. The initial excitation amplitude is assumed to be constant across all frequencies and proportional to the voltage (see Figure S1): $A_{0}=\beta U_{pp}$ which gives, for the fragmentation length:

$L_{frag}=\frac{1}{Im\left\{ k \right\}}\ln\left( \frac{R_{\mathrm{jet}}}{\beta U_{\mathrm{pp}}} \right)$ (3)

For a frequency of 1200 Hz, $L_{frag}$was measured as a function of ln(U_pp_). With no voltage applied to the jet, the slope was found to be equal to 9.63 × 10⁻⁴ m corresponding to a growth rate of 1038 m⁻¹. The normalization coefficient is then determined by multiplying the growth rate found by the fragmentation length corresponding to the $U_{\mathrm{pp}}$. We found it to be equal to 10.51. Figure S7C finally shows the growth rate obtained as a function of excitation frequency.

Using fragmentation length to measure growth rate suffers from certain limitations. Indeed, the transient regime at the onset of the jet and the nonlinear regime before the jet breaks up are not taken into account. Similarly to Gonzalez and Garcia [36], three subdomains of the jet can thus be defined: a linear transient part with length $L_{lt}$​, a linear purely exponential part with length $L_{le}$​, and a final nonlinear part with length $L_{nl}$. The fragmentation length is therefore equal to the sum of the lengths of these three regimes: $L_{frag}=L_{lt}+L_{le}+L_{nl}$.


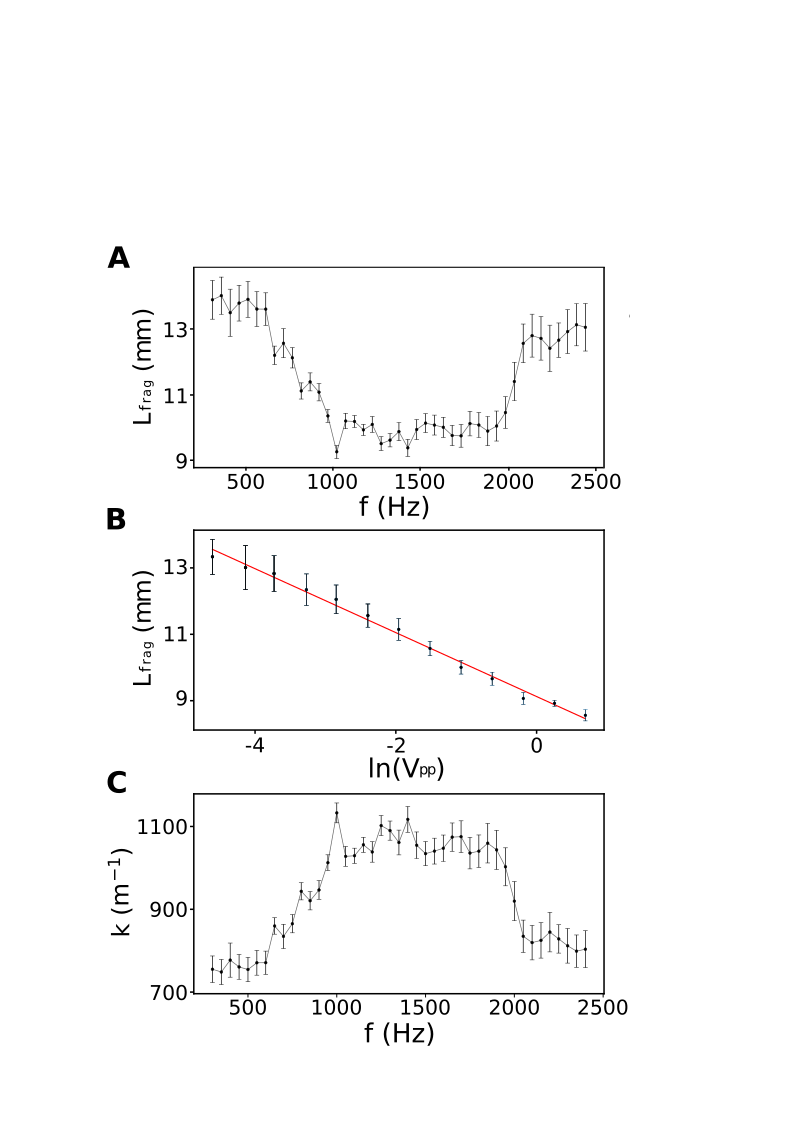
Near the injector (z=0), the perturbation amplitude does not grow exponentially: this is the initial linear transient part with a length $L_{lt}$. Near the fragmentation of the jet (z=L_frag_), the deformation of the jet becomes nonlinear over a length $L_{nl}$, with the development of higher-order harmonics. Thus, these two regimes do not affect the value of $\mathrm{Im}\left\{ k \right\}$ found during the linear regression at 1200Hz. However, the rest of the points of the dispersion curves are calculated from the fragmentation length $L_{frag}$ multiplied by the normalization coefficient $\ln\left( \frac{R_{jet}}{A_{0}} \right)$. These growth rates are therefore affected by the initial linear transient part and the final nonlinear part.

**Figure S7.** **Calculation of the growth rate.** (A) Fragmentation length as a function of the excitation frequency at *U_PP_* = 6V. (B) Fragmentation length as a function of the voltage applied to the piezoelectric actuator at *f* = 1200 Hz. (C) Growth rate as a function of excitation frequency at *U_PP_* = 6V.

**Figure S8**


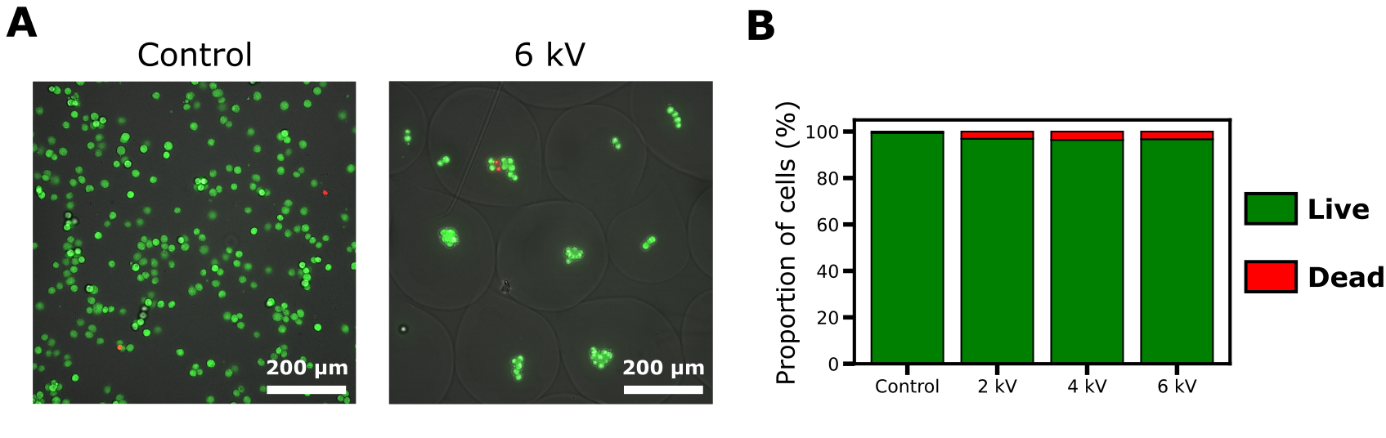


**Figure S8.** **Effect of the electric potential on the viability of induced pluripotent stem cells (iPSC).** (A) Live/dead staining of iPSC before and after encapsulation performed with U=6kV and no mechanical actuation. Images are obtained from a maximum intensity projection of a

z-stack captured with a spinning disk confocal microscope. (B) Bar chart illustrating the proportion of live/dead iPSC cells before encapsulation (control, survival rate=99.4%, N=1244), for U=2 kV (survival rate=96.9%, N=228), U=4 kV (survival rate=96.4%, N=168) and for U=6 kV (survival rate=96.7 %, N=123).
